# Effects of Catastrophic Coverage Expansion on Out-of-Pocket Spending for Non-Covered Services and Financial Equity: Evidence from South Korea’s National Health Insurance

**DOI:** 10.3390/healthcare14030302

**Published:** 2026-01-26

**Authors:** Minjeong Kim, Donggyo Shin, Hyunwoung Shin, Jangho Yoon

**Affiliations:** 1 Institute for Innovation in Digital Healthcare, Yonsei University Health System, 50-1, Yonsei-ro, Seodaemun-gu, Seoul 03722, Republic of Korea; mjkim85@yuhs.ac; 2 Medical Record Department, Ilsan Hospital, 100, Ilsanro, Ilsandong-gu, Goyang-si 10444, Gyeonggi-do, Republic of Korea; 3 Center for Health Insurance Research, Department of Health Care Policy Research, Korea Institute for Health and Social Affairs, (Building D) 370 Sicheongdaero, Sejong-si 30147, Republic of Korea; shw1114@naver.com; 4 Division of Health Policy and Management, Department of Preventive Medicine & Biostatistics, F. Edward Hebert School of Medicine, Uniformed Services University of Health Sciences (USU), Bethesda, MD 20814, USA; jangho.yoon@usuhs.edu

**Keywords:** catastrophic healthcare benefits, non-covered services, financial protection, national health insurance, out-of-pocket spending, South Korea

## Abstract

**Background:** Patients with catastrophic health conditions have continuously faced substantial out-of-pocket (OOP) costs for non-covered services despite universal health coverage in South Korea. In 2013, the National Health Insurance Service (NHIS) expanded coverage for four major catastrophic conditions—cancers, cardiovascular diseases, cerebrovascular diseases, and rare illnesses—aiming to strengthen financial protection for patients with catastrophic conditions. However, concerns remain that providers may respond by inducing more use of non-covered services, potentially offsetting reductions in patients’ financial burden. **Methods:** We evaluated the impact of the 2013 catastrophic coverage expansion on patients’ OOP spending for non-covered services using a quasi-experimental difference-in-differences design. Using nationally representative longitudinal healthcare expenditure data, the Korean Health Panel Survey (KHPS), from 2011 to 2016, we compared patients with the four targeted conditions to a control group with clinically comparable conditions. A two-part model was applied to separately estimate changes in the probability of incurring any non-covered OOP spending and changes in spending levels conditional on positive expenditures. We further examined whether effects differed by supplemental private health insurance (PHI) status. **Results:** We found that 7.3-, 5.2-, and 7.7-percentage-point decreases in annual probability of incurring any non-covered OOP spending for total, inpatient, and outpatient services, respectively, after policy implementation. Among patients with positive spending, OOP spending for total and inpatient non-covered services decreased by approximately 164 USD and 254 USD per year, while outpatient spending showed no statistically significant change. No statistically significant differential effects were also observed by PHI status. **Conclusion:** The catastrophic coverage expansion reduced patients’ exposure to and burden of non-covered OOP spending, indicating improved financial protection without evidence of compensatory increases in non-covered service use. These findings suggest that targeted benefit expansions for high-cost conditions can enhance financial equity within universal health systems.

## 1. Background

Despite implementing universal health care through the national health insurance (NHI) system in 1989, South Korea continues to witness patients facing significant financial burdens for high-cost medical procedures raised from the limited scope of covered services [1]. In 2005, the government introduced the Mid-term Health Benefits Expansion Security Plan (MHBESP) to address this issue.

The MHBSP was phased progressively over three phases (2005, 2009, and 2013). During the first two phases (2005–2008, 2009–2012), the policy mainly expanded the benefits by reducing co-insurance rates (from 20% to 10% in 2005 and from 10% to 5% in 2009). Starting in 2013, the benefits were expanded by including healthcare services for four particular catastrophic conditions—cancers, cardiovascular diseases, cerebrovascular diseases, and rare illnesses—in the package to alleviate patients’ financial strain [1]. The four major catastrophic conditions are life-threatening and require expensive healthcare services, including anticancer medications, advanced diagnostics, and treatment technologies. As the healthcare spending associated with those conditions accounted for a substantial share of national health expenditures, the government prioritized the four conditions. More than 580 services were newly added to the coverage package in this phase (hereinafter referred to as the “catastrophic coverage expansion”), such as ultrasound, MRI, anticancer medications, radiography, advanced clinical examinations, and genetic tests, at costs of over 9 billion U.S. dollars (USD) for 5.5 million covered lives [2].

For a theoretical perspective, the catastrophic coverage expansion may affect patients’ out-of-pocket (OOP) spending for non-covered services through two competing mechanisms. On the supply side, supplier-induced demand (SID) theory suggests that providers facing reduced margins for newly covered services may compensate by increasing the provision of non-covered services, which remain unregulated and more profitable, potentially increasing patients’ OOP spending [3,4]. This concern is particularly salient in the Korean healthcare system, where approximately 94% of hospitals and clinics are privately owned, providers are reimbursed on a fee-for-service basis, and extensive government regulation of NHIS-covered services, while non-covered services are not [5]. Under these conditions, non-covered services, which are not subject to price regulation under the NHIS, can generate additional revenues for providers at the expense of greater patient financial liability. Therefore, the policy would lead to unchanged or higher OOP expenditures for non-covered services.

In contrast, on the demand side, healthcare services for catastrophic conditions are largely non-discretionary and characterized by low price elasticity of demand. Expanding coverage for previously non-covered essential services may therefore reduce patients’ reliance on substitute non-covered services, resulting in lower OOP spending. Under this mechanism, the coverage expansion would lead to a decline in spending on non-covered services rather than compensatory increases.

These theoretical considerations generate two competing empirical hypotheses: if supplier-induced demand dominates provider behavior, OOP spending for non-covered services would increase or remain unchanged following the policy; if patient-side mechanisms dominate, OOP spending for non-covered services would decline. This study empirically tests these competing hypotheses by examining changes in OOP spending for non-covered services following the 2013 catastrophic coverage expansion.

The impact of the catastrophic coverage expansion may also vary across subpopulations. Four in five NHIS enrollees subscribe supplemental coverage to mitigate the financial burden for non-covered healthcare services in South Korea [6]. Given the importance of supplemental health insurance coverage within an NHI system [7], we extended our analysis to explore potential heterogenous responses to the policy change by individuals with and without supplemental private health insurance (PHI). As supplemental PHI reimburses policyholders for their actual OOP costs including all non-covered services, it remains empirically unclear whether supplemental PHI might moderate the impact of the catastrophic coverage expansion on OOP expenditures for non-covered services.

Although several studies have examined changes in overall healthcare spending following benefit expansions in Korea, no prior study has directly focused on patients’ OOP spending for non-covered services attributable to the catastrophic coverage expansion. Addressing this gap is critical given the substantial role of non-covered services in shaping patient financial burden.

To address this gap, we employed a quasi-experimental difference-in-differences design using nationally representative healthcare expenditure survey data from 2011 to 2016. By focusing on out-of-pocket spending for non-covered services, this study provides empirical evidence on the financial protection effects of catastrophic coverage expansion and informs ongoing policy debates on benefit design within universal health insurance systems.

## 2. Methods

### 2.1. Data and Sample

The Korean Health Panel Survey (KHPS), jointly conducted by the Korean Institute for Health and Social Affairs (KIHASA) and the National Health Insurance Service (NHIS), provides comprehensive data on healthcare service utilization and expenditures among non-institutionalized civilians in South Korea [8]. The data retrieved from the KHPS contained annual healthcare expenditures by service type and comprehensive population characteristics, such as demographic and socio-economic factors, as well as self-assessed and validated health status and health behavior measures.

The sample included 8251 individuals (22,003 observations) followed annually from 2011 through 2016. We split the sample into two groups: the policy intervention group, consisting of 2697 individuals (32.7% of the sample), and the comparison group, consisting of 5554 individuals (67.3%). The policy intervention group consisted of individuals diagnosed with any of the four major catastrophic conditions (cancers, cardiovascular diseases, cerebrovascular diseases, and rare illnesses) over the study period, as defined by the ‘Guideline to Implement Legislation for Expanding Benefits Coverage for Critical and Rare Illnesses’ [9]. To construct a comparison group with healthcare utilization patterns comparable to those of the policy intervention group, we selected 17 medical conditions associated with high healthcare expenditures and substantial OOP payments for non-covered services. These conditions were identified using two nationally representative administrative sources: the 2012 Annual Survey on Medical Expenses of National Health Insurance Enrollees [10] and the Analysis on Patients of High-Paying and Critical Diseases among NHIS Enrollees [11]. From these sources, we selected conditions that (1) ranked among the highest in total medical expenditures and (2) were not included in the catastrophic coverage expansion implemented in 2013. This approach ensured that the comparison group was similar to the policy group in baseline spending intensity and exposure to non-covered services, while remaining unaffected by the policy intervention. The full list of conditions, corresponding disease codes, and data sources are reported in Supplementary Table S1. We restricted our analysis to NHIS enrollees, excluding Medical Aid beneficiaries from the sample.

### 2.2. Variables

Outcome variables included patients’ annual OOP costs for non-covered services, categorized separately into inpatient, outpatient, and total costs. Initially recorded in Korean won (₩), OOP costs were converted to USD at a rate of $1 = ₩1311. All expenditure variables were first adjusted for inflation using the Medical Care Consumer Price Index (CPI), with 2013 as the base year, and subsequently converted to U.S. dollars; all monetary values are reported in 2024 USD.

The main independent variables included a binary indicator for the policy intervention group, defined as individuals with the four catastrophic conditions, with the comparison group, consisting of individuals with the comparable 17 conditions, serving as the reference category. Additionally, a binary indicator for the post-policy intervention period (2013–2016) was included, with the pre-policy intervention period (2011–2012) as the reference category. Further, a binary indicator for PHI was also constructed.

We controlled for potential confounders using a modified Andersen Behavioral Model [12]. Covariates included predisposing factors (such as age, sex, educational level, marital status, the number of children, and residence areas), enabling factors (such as annual household income, employment status, and PHI), and need factors (such as chronic diseases, self-assessed health status, and disability status).

### 2.3. Econometric Analysis

#### 2.3.1. Two-Part Difference-in-Differences Approach

We employed a two-part model [4,13,14,15,16] within a difference-in-differences (DID) framework [17,18] to examine the impact of the catastrophic coverage expansion on out-of-pocket (OOP) spending for non-covered services. This modeling strategy is well suited for healthcare expenditure data, characterized by a large mass of zero observations [15] and a highly right-skewed distribution among positive values.

In the first part, we estimated the probability of incurring any positive OOP spending for non-covered services in a given year (i.e., zero versus non-zero expenditure), capturing the *extensive margin* of healthcare utilization—whether patients used any non-covered services at all. In the second part, conditional on having positive OOP spending, we estimated the level of OOP expenditures for non-covered services, capturing the *intensive margin*—the amount of spending among users of non-covered services as below:

(1) $$Part\;1:\mathrm{Pr}\left(y_{it}\right)=f({policy}_{i},\;{post}_{t},\;{policy}_{i}\times {post}_{t},\;x_{it}^{\prime },\;\mu_{i})$$

(2) $$Part\;2:(y_{it}|y_{it}>0)=f({policy}_{i},\;{post}_{t},\;{policy}_{i}\times {post}_{t},\;x_{it}^{\prime },\;\mu_{i})$$

where i denotes an individual, t indicates a year, and f represents a distribution function discussed below. The dependent variable y includes patients’ OOP costs for non-covered services (separately for inpatient, outpatient, and total). The interaction term between the policy intervention group (policy) and post-period indicators (post) is the main variable of interest as its coefficient may measure the effect of the catastrophic coverage expansion on each outcome. The regression model controlled for between-group heterogeneity (policy), overall pre-post time trend (post), and comprehensive individual-level confounders (x′). The unobserved heterogeneity ($\mu_{i}$) was also controlled for to further minimize bias due to selection on unobservables and was addressed by using time-demeaned data.

The two-part structure reflects important behavioral and institutional features of healthcare utilization. While the decision to initiate care is largely driven by patients, the intensity and cost of care after initiation are substantially influenced by physicians, who act as agents for patients and determine treatment intensity, service mix, and associated costs [17]. Because these two processes are governed by different mechanisms, estimating them jointly using a single-index model may lead to biased estimates. By modeling participation and intensity separately, the two-part model accommodates the semi-continuous nature of healthcare expenditure data, accounts for heterogeneity between zero and positive expenditures, and yields more reliable estimates in the presence of skewness and excess zeros.

In this study, zero out-of-pocket (OOP) spending for non-covered services may reflect either the absence of utilization of non-covered services or the receipt of healthcare services that were fully covered by the NHIS and therefore required no patient payment for non-covered items. Accordingly, the first part of the two-part model captures patients’ exposure to financial liability for non-covered care rather than overall healthcare utilization

To explore heterogeneity in the effects of the catastrophic coverage expansion between individuals with and without PHI (phi), we estimated a two-part triple-differences model as follows:

(3) $$Part\;1:\;Pr(y_{it})=f({policy}_{i},\;{post}_{t},\;{policy}_{i}\times {post}_{t},\;{policy}_{i}\times {post}_{t}\times {phi}_{i},\;x_{it}^{\prime })$$

(4) $$Part\;2:\left(y_{it}|y_{it}>0\right)=f({policy}_{i},\;{post}_{t},\;{policy}_{i}\times {post}_{t},\;{policy}_{i}\times {post}_{t}\times {phi}_{i},x_{it}^{\prime })$$

The coefficient on the interaction term policy×post now measures the effect of the catastrophic coverage expansion for individuals without PHI. The triple interaction term policy×post×phi is the variable of main interest, as its coefficient may capture an incremental effect of the catastrophic coverage expansion for individuals with PHI, compared to others without PHI. Thus, while the coefficient on policy×post assesses the policy’s impact on individuals without PHI, the coefficient on policy×post×phi evaluates the additional impact for those with PHI. We computed marginal effects as the linear combination of coefficients, separately for individuals with and without PHI.

The validity of the DID approach relies on the parallel-trends assumption, so we formally tested this assumption by examining whether pre-policy trends (2011–2012) in OOP spending for non-covered services differed between the policy and control group and we found no statistically significant differences (see Supplementary Table S2). In addition to the formal falsification tests, we also visually confirm that the pre-policy trends were parallel across groups using pre-policy trend plots that compare mean out-of-pocket spending for non-covered services between the policy and control groups during the pre-intervention period (2011–2012). These plots are presented in the Supplementary Materials (Supplementary Figures S1–S3).

#### 2.3.2. Estimation

We employed generalized linear models (GLMs) to estimate the two-part DID specifications. GLMs are appropriate for the healthcare expenditure data because they allow flexible combinations of link functions and variance structures to accommodate the right-skewed distribution of medical spending [15]. Model specification was guided by Box–Cox tests to inform the choice of link function and Modified Park tests to select the appropriate distribution family.

In the first part, we estimated the model using an identity link and Gaussian family, corresponding to a linear probability model (LPM). Although nonlinear models such as logit or probit are commonly used for binary outcomes, the LPM is widely applied in DID settings because the coefficient on the interaction term directly identifies the average treatment effect on the probability scale. This avoids the complications in interpreting interaction effects in nonlinear models and allows straightforward interpretation of estimates as percentage-point changes in probability. Further, because our interest lies in estimating average policy effects rather than individual-level prediction, the LPM provides a transparent and interpretable approach for estimating changes in the likelihood of incurring non-covered OOP spending following the catastrophic coverage expansion.

In the second part of the model, conditional on positive spending, Box–Cox diagnostics indicated log-type mean functions for all expenditure outcomes, with estimated power parameters close to zero for total (δ = 0.07) and outpatient (δ = 0.09) OOP spending, and slightly higher for inpatient spending (δ = 0.17). Modified Park tests suggested variance–mean relationships consistent with a Gaussian distribution for total and outpatient spending (coefficients ≈ 0.08, 0.09, respectively), while inpatient spending showed modest overdispersion (coefficient ≈ 0.55). Although Box–Cox results favored a log link, log-link specifications exhibited convergence instability in this dataset. We therefore employed power link functions that approximate log-type relationships while ensuring numerical stability (power 0.3 for total, 0.2 for inpatient, and 0.5 for outpatient spending), in combination with a Gaussian family. These diagnostic results are summarized in Supplementary Table S5.

We did not apply formal adjustments for multiple testing. The outcomes examined—total, inpatient, and outpatient OOP spending for non-covered services—were specified a priori and represent closely related dimensions of a single policy-relevant construct, namely patients’ financial burden. Similarly, alternative model specifications and subgroup analyses by supplemental private health insurance status were motivated by theoretical considerations rather than exploratory testing. Consistent with common practice in DID policy evaluations, we therefore report unadjusted *p*-values and interpret findings based on the consistency and magnitude of effects across outcomes.

DID effects were captured by the interaction between the policy group indicator and the post-policy period indicator. All models included year fixed effects and covariates capturing social, economic, health status, and private insurance characteristics. All standard errors were clustered at the individual level to account for within-person correlation in the longitudinal panel data. Individual fixed effects were implemented using a demeaning (first-difference) approach rather than a within-transformation. Specifically, outcome and covariate variables were transformed by subtracting individual-specific means prior to estimation, and the transformed variables were then estimated using the glm command. This approach yields estimates equivalent to fixed-effects estimation while allowing consistent implementation within the two-part modeling framework.

All analyses were conducted using STATA (version 15). The two-part DID models were estimated using STATA’s glm command.

## 3. Results

### 3.1. Population Characteristics

Table 1 presents the characteristics of the study population, separately for the policy intervention and control groups in the pre-intervention periods. Baseline differences are assessed using both *p*-values and standardized mean differences (SMDs). We employed the SMDs to assess the magnitude of baseline differences in this observational setting, which are less sensitive to sample size than hypothesis tests. With *p*-values, the two groups had no significant differences in most observed baseline characteristics. Although some group differences in observable baseline characteristics were statistically significant, their magnitudes were often small and of limited practical significance. For instance, the mean ages for the policy intervention and comparison groups were 62.0 and 63.7, respectively. Gender distribution was also similar, with females comprising 59% of the policy intervention group and 61.7% of the comparison group. The average number of chronic conditions was 4.0 in the policy intervention group compared to 3.9 in the comparison group. The other baseline characteristics, including education, marital status, place of residence, household income, employment status, and perceived health, were also broadly comparable between the groups. With SMDs, they also indicate that most imbalances are small to moderate in magnitude, suggesting reasonable baseline comparability between the groups. Concerning outcome measures, prior to the policy implementation, patients with catastrophic conditions exhibited substantially higher total and inpatient OOP spending for non-covered services than the control group, underscoring their disproportionate financial burden. This pattern is consistent with the high-cost, inpatient-centered nature of care for life-threatening conditions. 

### 3.2. Changes in OOP Spending for Non-Covered Services Associated with the Benefits Expansion

Table 2 reports the results from the two-part DID regression models on the time-demeaned data. The coefficients on the interaction term, policy × post, are of main interest as they capture DID estimates of the effects of the benefits expansion. In Part 1, it was −0.073 (*p* < 0.001), suggesting that the implementation of the catastrophic benefits expansion was attributable to a 7.3-percentage-point decrease in patients’ exposure to financial risk at the extensive margin. In part 2, the coefficient on the interaction term indicates the benefits expansion was associated with a reduction of 164 USD in the yearly total OOP spending among those with positive total OOP spending (*p* = 0.023), which reflects a substantial alleviation of financial burden. These outcomes indicate that the policy not only reduced exposure to non-covered services but also lowered the depth of financial liability among users.

Overall, the catastrophic coverage expansion was associated with an approximate reduction of USD 139 per person-year in expected total OOP spending for non-covered services. This represents a nontrivial share of baseline non-covered expenditures, which averaged approximately USD 390 per person-year in the pre-policy period, corresponding to a reduction of roughly 35%

The coefficients on inpatient OOP spending for non-covered services were qualitatively in line with those for total OOP spending, indicating a consistent pattern of negative relationship. The coverage expansion was associated with a 5.2-percentage-point decrease in the probability of incurring inpatient OOP spending (*p* = 0.004), and then a decrease of 254 USD for yearly inpatient OOP spending among those with any positive inpatient OOP spending (*p* = 0.021). These results underscore the promising impact of the catastrophic coverage expansion on inpatient OOP spending for non-covered services.

For outpatient OOP spending, the coverage expansion was associated with a 7.7-percentage-point decrease in the probability of incurring any outpatient OOP spending (*p* < 0.000) and increased outpatient OOP spending, on average, by 18 USD after the coverage expansion. However, it was not a statistically discernable change.

For catastrophic health conditions—where care is medically necessary, recurrent, and often high-cost—even moderate absolute reductions in OOP liability can substantially reduce the risk of financial hardship. Given that non-covered services have continuously accounted for a sizable share of patient financial responsibility in South Korea, the observed reduction of approximately USD 140 per year represents a considerable improvement in financial protection.

These findings also indicate that the catastrophic coverage expansion improved both the breadth and depth of financial protection following the catastrophic coverage expansion, indicating that the policy improved financial protection by reducing patients’ exposure to, and burden from, non-covered healthcare costs.

Table 2 shows that the catastrophic coverage expansion was associated with a lower probability of incurring any out-of-pocket spending for non-covered services, particularly for total and inpatient care, and with reduced spending among those who incurred such costs. In contrast, changes in outpatient spending intensity were small and not statistically significant

### 3.3. Heterogeneity by Supplemental PHI

**Figure 1:**
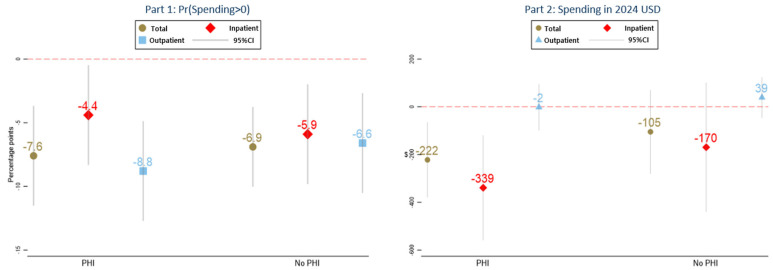
Heterogeneous responses to the catastrophic coverage expansion by individuals with and without supplemental PHI coverage: Marginal effects from the triple-interaction model. Note. Reported are marginal effects of the catastrophic coverage expansion for PHI and non-PHI subgroups. Interaction terms between policy exposure and PHI status were not statistically significant, indicating no detectable differential effect by PHI coverage.

Figure 1 depicts the results of a test for whether PHI coverage is linked to heterogeneous responses to the catastrophic coverage expansion for individuals with critical conditions. The marginal effects of the catastrophic coverage expansion are reported separately for the PHI and non-PHI subgroups, calculated using the estimated coefficients reported in Supplementary Table S3.

For individuals with PHI, the part 1 results show that the catastrophic coverage expansion was associated with 7.6-, 4.4-, and 8.8-percentage-point decreases in the annual probability of incurring positive total, inpatient, and outpatient OOP costs, respectively. The part 2 estimates indicate that OOP costs for total and inpatient non-covered services decreased by 222 USD and 339 USD, respectively.

For individuals without PHI, the probability of incurring positive non-covered OOP spending decreased by approximately 6.9, 5.9, and 6.6 percentage points for total, inpatient, and outpatient services, respectively, and decreased by 105 USD and 170 USD in total and inpatient OOP spending for non-covered services. A small, positive marginal effect was found for outpatient OOP spending.

When results are stratified by supplemental PHI status, the estimated effects of the catastrophic coverage expansion on OOP spending for non-covered services are broadly similar in direction for individuals with and without PHI. These patterns suggest that the catastrophic coverage expansion reduced both exposure to and intensity of non-covered healthcare spending for patients regardless of supplemental insurance status. The larger point estimates observed among individuals with PHI may reflect differences in baseline utilization patterns or the scope of services reimbursed by PHI, but the absence of statistically significant interaction effects indicates that these differences should be interpreted cautiously. The interaction terms between the policy indicator and supplemental PHI status were not statistically significant for any of the main outcomes, indicating no detectable differential effect of the catastrophic coverage expansion by PHI status.

## 4. Discussion

This study examined whether the 2013 catastrophic coverage expansion under the Korean National Health Insurance Service (NHIS) influenced patients’ out-of-pocket (OOP) spending for non-covered services. Our findings indicate that the policy was associated with a significant reduction in OOP costs for non-covered services among individuals with catastrophic conditions. Following the policy implementation, the probability of incurring any OOP spending for non-covered services declined by 7.3, 5.2, and 7.7 percentage points for total, inpatient, and outpatient services, respectively. Conditional on having any OOP spending, total and inpatient OOP expenditures for non-covered services decreased by USD 164 and USD 254, respectively, while changes in outpatient spending intensity were not statistically significant.

The magnitude of the estimated reductions is substantial when viewed relative to baseline spending levels. Prior to the policy, patients with catastrophic conditions incurred an average of USD 568 per year in total out-of-pocket payments for non-covered services, including USD 343 for inpatient care. Against this baseline, the estimated reductions of USD 164 in total spending and USD 254 in inpatient spending among those with positive expenditures correspond to sizable declines in financial burden. In particular, the inpatient effect represents a large share of baseline inpatient out-of-pocket costs, underscoring the policy’s relevance for alleviating high-cost hospital-based financial exposure among patients with catastrophic conditions. Therefore, these results suggest that the catastrophic coverage expansion reduced both patients’ exposure to non-covered healthcare costs and the financial burden among those who continued to incur such expenses.

The absence of statistically significant effects for outpatient spending likely reflects the inpatient-centered nature of care for catastrophic conditions. Patients with cancers, cardiovascular diseases, cerebrovascular diseases, and rare illnesses primarily receive high-intensity treatment in inpatient settings, where both utilization and financial burden are concentrated. Accordingly, the financial protection effects of the catastrophic coverage expansion are more evident for inpatient services than for outpatient care.

From a health policy perspective, the structure and magnitude of these effects are noteworthy. The reduction in the probability of any non-covered OOP spending reflects improved financial protection at the extensive margin, indicating that fewer patients faced financial liability for services outside the NHIS benefit package. At the same time, the decline in spending among users of non-covered services points to reduced intensity of financial burden. Distinguishing between these two margins is important for policy evaluation, as reductions in exposure and in spending intensity represent different but complementary dimensions of financial protection. In the context of catastrophic health conditions—where care is medically necessary, recurrent, and often high-cost—even moderate absolute reductions in OOP spending may significantly reduce the risk of financial hardship.

These findings contribute to the existing literature on benefit expansion and supplier-induced demand. Prior studies of Korea’s health insurance reforms have primarily focused on changes in total medical expenditures or utilization of covered services, with mixed evidence regarding whether providers respond to coverage expansion by inducing demand for non-covered services. In contrast, our study directly examines OOP spending for non-covered services and finds no evidence of compensatory increases following the catastrophic coverage expansion. This pattern is consistent with theoretical and empirical work emphasizing the low-price elasticity of demand for medically necessary care in life-threatening conditions, and suggests that expanding coverage for catastrophic illnesses need not lead to cost shifting toward non-covered services.

Our analysis further shows that reductions in OOP spending for non-covered services were statistically similar for individuals with and without PHI. Although individuals without PHI tend to be more medically and socially vulnerable, we did not detect statistically significant differential effects by PHI status. This suggests that the catastrophic coverage expansion was associated with broadly similar financial protection effects across PHI groups.

Several limitations should be considered when interpreting these findings. First, the pre-intervention period is limited to two years (2011–2012), which constrains the ability to assess longer-term pre-policy trends. Although formal falsification tests did not indicate differential pre-policy trends between the policy and comparison groups, the presence of unobserved longer-term trends cannot be fully ruled out. Second, the policy examined represents the third phase of the Mid-term Health Benefits Security Plan, and residual effects from earlier phases may have carried over into the study period. Third, minor discrepancies between disease coding systems in the survey data and NHIS guidelines may have resulted in some misclassification of individuals into the policy or comparison groups. These limitations suggest that the findings should be interpreted as evidence of changes in financial burden associated with the policy, rather than definitive estimates of its long-term or system-wide effects.

In addition, our analysis cannot fully account for unobserved heterogeneity related to clinical severity, disease stage, or patient preferences. Although we control for observable health status indicators, patients with more severe disease or different treatment preferences may systematically differ in their use of non-covered services. If such unobserved factors vary over time or differentially across groups, they could influence both the likelihood and intensity of non-covered spending. Accordingly, our findings should be interpreted as average effects of the policy on out-of-pocket spending, rather than as precise estimates for specific clinical subgroups.

Within these constraints, our results provide evidence that the catastrophic coverage expansion was associated with meaningful reductions in OOP spending for non-covered services among patients with high-cost, life-threatening conditions. The findings support the role of targeted benefit expansions as a financial protection policy while underscoring the importance of continued monitoring to assess longer-term impacts and potential unintended consequences.

## 5. Conclusions

The 2013 coverage expansion for four catastrophic conditions—cancers, cardiovascular diseases, cerebrovascular diseases, and rare illnesses—was associated with reductions in out-of-pocket spending for non-covered services among affected patients in South Korea. These reductions reflected both a lower likelihood of incurring any non-covered OOP spending and lower spending levels among those who continued to incur such costs. The estimated effects were concentrated in inpatient care and were broadly similar for individuals with and without supplemental private health insurance.

These findings suggest that targeted catastrophic benefit expansions can enhance financial protection for patients with life-threatening conditions without evidence of compensatory increases in non-covered spending. While the policy appears to have reduced patients’ financial burden, further research is warranted to evaluate its long-term effects, cost-effectiveness, and potential implications for healthcare utilization and equity.

## Figures and Tables

**Table 1:** Characteristics of the Study Sample.

Variables	Definitions	Pre-Policy Periods (Baseline, 2011–2012)			
		Policy (*n* = 1840)	Control (*n* = 3691)	*p*-Values/χ^2^	SMD ^4^
**Explanatory Variables**					
*Social Demographic Factors*					
Age ^1^	the average age	62.0 (14.1)	63.7 (12.6)	4.40 *** ^3^	−0.13
Female	=1 if female; 0 otherwise	58.8%	61.7%	4.45 *	0.06
Marital Status	=1 if married; 0 otherwise	79.7%	74.9%	16.2 ***	0.12
Children ^1^	An average number of children	0.88 (1.0)	0.82 (1.1)	−1.79	0.06
Residence	=1 if living in Seoul (Capital)	14.7%	13.4%	1.59	0.04
*Education (Reference: < High school)*					
High School	=1 if a high school graduate	44.6%	34.2%	61.7 ***	0.21
College	=1 if have college or above	15.8%	11.4%		0.13
*Economic Factors*					
House income ^1,2^	Average yearly household income	$27,113.7 (24,686.5)	$24,718.6 (20,664.6)	−3.58 ***	0.11
Employment	=1 if currently employed	42.7%	50.2%	28.2 ***	−0.15
*Health Status Factors*					
Chronic Diseases ^1^	Number of chronic diseases	4.0 (2.5)	3.9 (2.3)	−1.36	0.04
*Perceived Health Status (PHS) (Reference: Very Bad)*					
Good	=1 if PHS is very good or good	21.6%	26.7%	16.9 ***	−0.12
Fair	=1 if PHS is fair	35.5%	41.1%	16.1 ***	−0.11
Bad	=1 if PHS is very bad or bad	33.9%	25.9%	38.4 ***	0.18
*Private Health Insurance (PHI)*					
PHI	=1 if have any PHI	53.3%	53.9%	0.14	−0.01
**Outcomes**					
*Out-of-pocket (OOP) payments for non-covered services * ^1,2^					
Outpatient services		$225.07 (523.37)	$155.87 (412.93)	−7.30 ***	0.15
Inpatient services		$342.83 (1202.76)	$145.22 (629.03)	−9.80 ***	0.21
Total OOP payments		$567.91 (1208.08)	$301.09 (787.86)	−11.5 ***	0.26
*Utilizations * ^1^					
Number of outpatient visits		33.7 (34.7)	35.1 (33.6)	1.44	−0.04
Hospital inpatient days		8.3 (24.5)	4.4 (20.1)	−6.0 ***	0.17

^1^ Mean (standard error). ^2^ KRW (Korean Currency) was converted to USD at a rate of $1 = ₩1311. ^3^ * *p* < 0.05, *** *p* < 0.001. ^4^ SMD denotes standardized mean difference, calculated as the difference in means (or proportions) divided by the pooled standard deviation. Absolute SMD values below 0.10 are typically considered negligible, values between 0.10 and 0.20 small, and values above 0.20 moderate. Baseline differences are expected in observational settings; identification relies on the parallel-trends assumption rather than equality of baseline levels.

**Table 2:** Changes in OOP spending on non-covered services attributable to the catastrophic coverage expansion: Coefficients from the two-part difference-in-differences models.

	Total OOP Spending		Inpatient OOP Spending		Outpatient OOP Spending	
	Part 1	Part 2	Part 1	Part 2	Part 1	Part 2
Policy	0.153 ***	291.27 ***	0.207 ***	298.12 **	0.157 ***	−29.67
	(0.015)	(71.30)	(0.018)	(100.03)	(0.016)	(43.39)
Post	0.118 ***	230.33	0.052 ***	35.27	0.126 ***	8.41
	(0.013)	(119.31)	(0.013)	(204.6535)	(0.013)	(66.59)
PolicyXPost	−0.072 ***	−163.68 *	−0.052 **	−254.07 *	−0.077 ***	17.91
	(0.015)	(72.05)	(0.018)	(110.22)	(0.015)	(40.38)
Age	0.007	−35.70	0.011 *	34.05	0.004	4.37
	(0.004)	(36.47)	(0.005)	(63.43)	(0.004)	(17.57)
High School	−0.003	207.10	0.139	−287.65	−0.019	44.80
	(0.055)	(203.68)	(0.0829)	(401.73)	(0.060)	(105.61)
College	0.019	−768.82	−0.035	−1103.09	0.021	−0.86
	(0.072)	(433.80)	(0.079)	(800.82)	(0.071)	(108.46)
Marital Status	−0.014	−158.33	−0.031	−11.98	−0.031	−127.88
	(0.030)	(133.63)	(0.029)	(146.57)	(0.031)	(110.57)
Children	0.007	−18.47	0.007	−5.33	0.005	−26.84
	(0.010)	(41.75)	(0.012)	(68.49)	(0.010)	(19.77)
Residence	0.009	−130.43	0.087	−500.17	0.024	−76.49
	(0.038)	(390.67)	(0.057)	(940.12)	(0.043)	(101.03)
House Income	0.000	−0.00	−0.000	−0.00	0.000	0.00
	(0.000)	(0.00)	(0.000)	(0.00)	(0.000)	(0.00)
Employment	0.004	−26.71	0.004	−93.01	0.085	24.00
	(0.011)	(53.90)	(0.013)	(84.21)	(0.012)	(27.65)
Chronic Diseases	0.001	−7.86	−0.005	−18.94	0.001	1.27
	(0.002)	(15.09)	(0.003)	(26.19)	(0.002)	(7.06)
Perceived Health_Good	−0.020 *	6.37	−0.005	23.18	−0.022 *	−7.45
	(0.009)	(32.86)	(0.019)	(57.87)	(0.009)	(21.52)
Perceived Health_Bad	0.018 *	48.37	0.045 ***	57.18	0.016 *	−7.09
	(0.008)	(37.05)	(0.010)	(56.33)	(0.008)	(21.81)
BMI	0.004 **	−2.49	−0.004 **	−4.11	0.009 ***	−0.72
	(0.001)	(5.12)	(0.001)	(6.59)	(0.002)	(2.25)
Disablity	−0.029	349.15	−0.088 *	624.38 *	−0.014	66.13
	(0.023)	(208.91)	(0.034)	(289.25)	(0.032)	(63.43)
PHI	0.008	−19.29	−0.002	106.04	0.027	−70.00
	(0.023)	(78.09)	(0.024)	(119.66)	(0.024)	(54.18)
Year 2012	0.093 ***	16.73	0.016	−27.46	0.099 ***	18.70
	(0.009)	(56.95)	(0.009)	(95.09)	(0.009)	(30.24)
Year 2013	0.046 ***	−123.99	−0.007	−32.07	0.047 ***	9.33
	(0.008)	(92.49)	(0.009)	(155.82)	(0.008)	(48.13)
Year 2014	0.039 ***	−110.07	0.005	1.91	0.042 ***	−40.81
	(0.005)	(57.32)	(0.006)	(92.96)	(0.005)	(30.71)
Year 2015	0.029 ***	−65.04	0.011	−30.06	0.031 ***	−31.60
	(0.005)	(58.74)	(0.006)	(93.87)	(0.006)	(29.45)
Year 2016	0.036 ***	46.89	−0.004	−14.63	0.036 ***	4.50
	(0.005)	(78.67)	(0.006)	(130.09)	(0.005)	(39.02)
_cons	−0.039 ***	551.03 ***	−0.004	689.52 ***	−0.041 ***	258.75 ***
	(0.004)	(52.41)	(0.004)	(84.98)	(0.004)	(26.35)
Total	18,908	6208	18,908	2855	18,908	6277

Note. Standard errors are in parentheses. All models controlled for person fixed-effects. * *p* < 0.05, ** *p* < 0.01, *** *p* < 0.001.

## Data Availability

The data that support the findings of this study is owned by the Korea Health Panel Study (KHPS). Data is publicly available upon permission from the authority. Users need to download the ‘Korea Health Panel Survey Data User Agreement’ form to obtain the data and send it back to ‘khp@kihasa.re.kr’. The form can be downloaded at https://www.khp.re.kr:444/eng/main.do (accessed on 1 September 2019).

## Supplementary Materials

The following supporting information can be downloaded at https://www.mdpi.com/article/10.3390/healthcare14030302/s1, Table S1: 17 Diseases Selected to Comprise the Control Group; Table S2: Common Parallel Assumption Tests; Table S3: Heterogeneous responses to the benefits expansion by supplemental PHI; Table S4: Healthcare services remained non-covered after 2013; Table S5: Model Diagnostic Test Results; Figure S1: A Pre-Period Trend of Non-Covered Total OOP Spending; Figure S2: A Pre-Period Trend of Non-Covered Inpatient OOP Spending; Spending; Figure S3: A Pre-Period Trend of Non-Covered Outpatient OOP Spending.

## Abbreviations

DID	Difference-in-Differences
KHPS	Korean Healthcare Panel Survey
KIHASA	Korean Institute for Health and Social Affairs
MHBSP	Mid-term Health Benefits Security Plan
NHIS	National Health Insurance Service
OOP	Out-of-Pocket
PHI	Private Health Insurance
SID	Supplier-Induced Demand
