# Supplementary material for: Effects of Catastrophic Coverage Expansion on Out-of-Pocket Spending for Non-Covered Services and Financial Equity: Evidence from South Korea’s National Health Insurance
Source: Healthcare (Basel). 2026 Jan 26;14(3):302. doi: 10.3390/healthcare14030302 (PMC12896595; doi:10.3390/healthcare14030302)
Supplement: Supplementary file 1 [file healthcare-14-00302-s001.zip › healthcare-4055774-supplementary.pdf]

[Supplementary Appendix]

1. Title: Effects of Catastrophic Coverage Expansion on Out-of-Pocket Spending for Non-covered Services and Financial Equity: Evidence from South Korea's National Health Insurance

2. Journal name: Healthcare

3. Authors' name: Minjeong Kim, Donggyo Shin, Hyunwoung Shin, Jangho Yoon (PhD)

4. Corresponding author's affiliation and email address

Name: Donggyo Shin

Affiliation: Medical Record Department, Ilsan Hospital, 100, Ilsanro, Ilsandong-gu, Goyang-si 10444, Gyeonggi-do, Republic of Korea

Country: South Korea

Email: donggyo@nhimc.or.kr

ORCID details: 0000-0001-8619-347X

1. 17 Diseases to Comprise a Control Group

To comprise a control group, comparable to a policy group in terms of OOP payments for non-covered services, we consulted the '2012 Annual Survey on Medical Expense of National Health Insurance Enrollees (NHISC, 2013)' to identify the top 50 conditions that caused high medical expenses in 2012. We selected 12 conditions that would match the four catastrophic conditions newly-covered for the policy intervention group. We also used the 'Analysis on Patients of High-Paying and Critical Diseases among NHI Enrollees (2005)' (NHISC, 2007) to add five additional conditions, which led to a total of 17 conditions being selected to define the comparison group.

< Supplementary Table S1 > 17 Diseases Selected to Comprise the Control Group

No.	KCD-6 Disease Codes	Diagnoses	Reference
1	A41	septicemic	Annual Survey on Medical Expense of National Health Insurance Enrollees (NHIC, 2013)
2	A55-56	Sexually transmitted diseases due to Chlamydia trachomatis	
3	A86	Viral meningoencephalitis NOS	
4	B20-B24	Human immunodeficiency virus (HIV) disease	
5	B05	measles	
6	F03	Unspecified dementia	
7	F10	Mental and behavioral disorders due to harmful use of alcohol	
8	G80-G83	Cerebral palsy and other paralytic syndromes	
9	N17, N19	Renal failure (N18 excluded)	
10	P05-P07	Disorders related length of gestation and fetal growth	
11	P28	Primary failure to expand terminal respiratory units	
12	S72	Fracture of femur	
13	M51	Other intervertebral disc disorders	Analysis on Patients of High-Paying and Critical Diseases among NHI Enrollees (NHIC, 2007)
14	M48	Other spondylopathies	
15	M17	Gonarthrosis [arthrosis of knee]	
16	M25	Senile cataract	
17	E11	Non-insulin-dependent diabetes mellitus	

2. Falsification Analysis

The DID estimation is only validated under the common parallel condition in pre-periods. We implemented the falsification test to inspect whether the policy and the control group have a parallel time trend in pre-periods.

For the falsification test, we applied the equation below,

$$y_{it} = \beta_0 + \beta_1\ policy_i + \beta_2\ year_t +, \beta_3\ policy_i \times year_t, + x'_{it} + \mu_i$$

where i and t indicate an individual and a year, respectively. The dependent variable y refers to patients' total OOP payments for non-covered services. The year variable (year) in this equation indicates pre-policy periods (2011-2012). The interaction term of the policy group and the year ($policy_i \times year_t$) is a main variable of interest. Its coefficient may capture differences between the policy and control groups in pre-policy periods.

In the Supplemental Table S2, the p-value (p= 0.340) of the interaction terms displays insignificance at any significance level for total spending of non-covered services, so that we can assume that the policy and control group have similar pre-period trend of total OOP costs for non-covered services. We can also assume that no other unobserved factors might affect to estimate the effects of the policy change on total costs for non-covered services through the DID estimation.

P-values of other outcomes (OOP for non-covered inpatient services, outpatient services, stays in hospitals, and visits to hospitals) are also statistically insignificant at any levels (include p-values). Therefore, we can assume that there are no statistically significant differences between the policy and the control groups in pre-policy periods.

We also provide graphical evidence showing pre-policy trends of the policy and control groups regarding non-covered total, inpatient, and outpatient OOP spending to

confirm the parallel trends assumption underlying the DID design (Supplementary Figures S1–S3).

< Supplementary Figure S1 > A Pre-Period Trend of Non-Covered Total OOP Spending

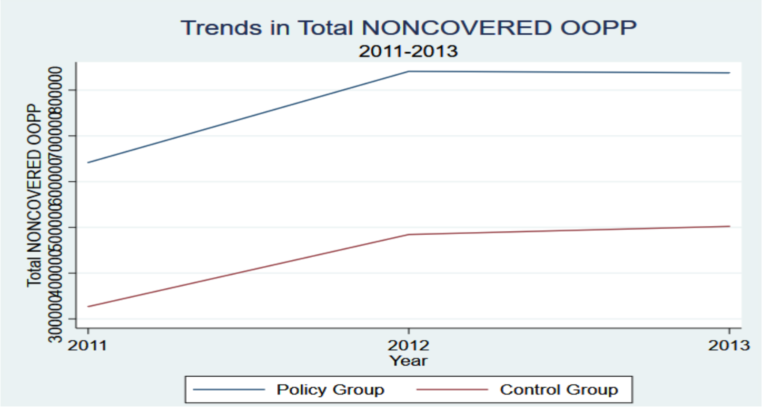

< Supplementary Figure S2 > A Pre-Period Trend of Non-Covered Inpatient OOP Spendings

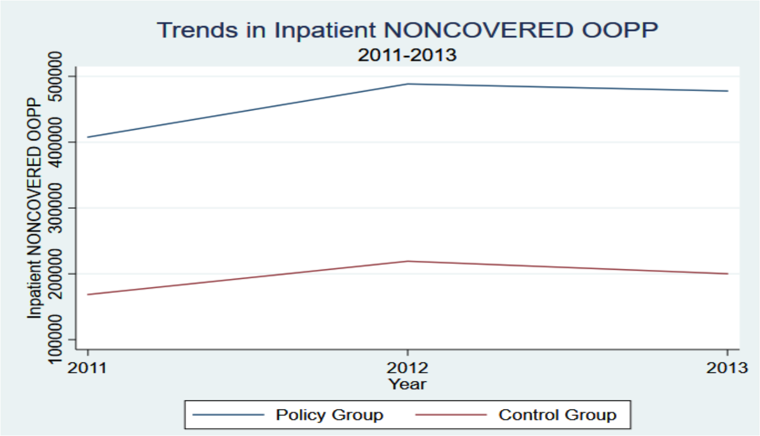

< Supplementary Figure S3 > A Pre-Period Trend of Non-Covered Outpatient OOP

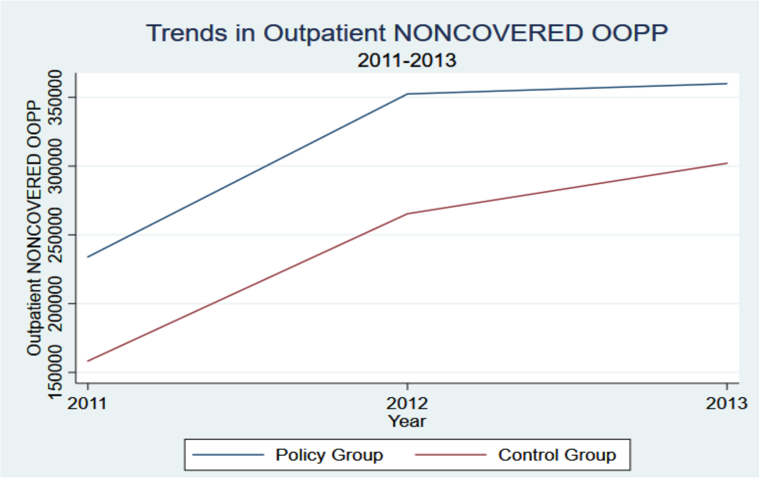

Note. Pre-policy trends in total, inpatient, and outpatient out-of-pocket spending for non-covered services, 2011-2012. The figures show mean annual spending for the policy and control groups prior to the 2013 catastrophic overage expansion

< Supplementary Table S2 > Common Parallel Assumption Tests

Spending for non-covered services	Total	Inpatient Services	Outpatient Services	Days of Visits	Stays in Hospitals
Year(Pre-Policy)	74.63***	11.71	62.92***	1.37	-2.19
	(21.35)	(15.71)	(13.28)	(1.13)	(2.18)
Policy	-119943.37	-77874.96	-42068.40	-410.77	-5052.19
	(125861.99)	(107192.09)	(54263.39)	(3845.96)	(7321.03)
Policy#Year	59.77	38.82	20.95	0.20	2.51
	(62.57)	(53.29)	(26.98)	(1.91)	(3.64)
Age	-5.20**	-3.44*	-1.76*	0.16*	-0.04
	(1.80)	(1.52)	(0.72)	(0.07)	(0.12)
Female	-44.01	-30.06	-13.95	0.59	-4.17
	(28.14)	(21.13)	(16.00)	(1.48)	(2.35)
High School	-13.51	-4.52	-8.99	-3.99**	1.87
	(29.72)	(24.05)	(14.82)	(1.41)	(3.95)
College	95.87	101.93	-6.06	-2.85	-3.09
	(63.67)	(54.10)	(24.13)	(1.75)	(3.56)
Marital Status	-3.18	-6.35	3.17	-0.54	-5.84
	(32.60)	(26.70)	(15.37)	(1.70)	(4.01)
No. Children	-35.92*	-9.91	-26.02***	0.01	0.85
	(15.13)	(12.60)	(6.36)	(0.70)	(1.01)
Residence	40.30	-19.18	59.48**	-3.21*	-3.15
	(38.88)	(29.71)	(19.65)	(1.51)	(5.12)

House Income	0.00<br>(0.00)	0.00<br>(0.00)	0.00** (0.00)	-0.00<br>(0.00)	-0.00* (0.00)
Employment	-28.92<br>(28.07)	-36.40<br>(22.86)	7.48<br>(13.94)	-0.18<br>(1.28)	-5.43* (2.18)
No. Chronic Diseases	7.84<br>(5.43)	-0.16<br>(4.43)	8.00** (2.60)	4.15*** (0.34)	-0.19<br>(0.44)
Perceived Health _Good	-53.71* (25.95)	-29.31<br>(20.88)	-24.39<br>(13.92)	1.27<br>(1.33)	-5.51* (2.63)
Perceived Health _Bad	115.07*** (32.92)	88.19** (27.13)	26.88<br>(15.94)	9.43*** (1.53)	3.75<br>(2.86)
BMI	-4.75<br>(2.58)	-5.21* (2.28)	0.46<br>(1.06)	0.07<br>(0.16)	-0.53* (0.24)
Disability	29.20<br>(54.18)	47.69<br>(48.41)	-18.49<br>(20.28)	4.26<br>(2.62)	9.01<br>(4.67)
Private Health Insurance	46.78<br>(26.85)	29.28<br>(19.85)	17.50<br>(15.88)	-3.45* (1.52)	2.76<br>(2.75)
_cons	-149473.30*** (42939.41)	-23108.98<br>(31589.65)	-126364.32*** (26702.61)	-2753.35<br>(2273.85)	4448.17<br>(4390.77)
Total	5260	5260	5260	3571	983

Standard errors in parentheses
* $p < 0.05$, ** $p < 0.01$, *** $p < 0.001$

3. Heterogeneous responses to the catastrophic coverage expansion by individuals with and without supplemental PHI coverage: Coefficients from the triple-interaction model.

< Supplementary Table S3 > Heterogeneous responses to the benefits expansion by supplemental PHI

	Total OOP spending		Inpatient OOP spending		Outpatient OOP spending	
	Part 1	Part 2	Part 1	Part 2	Part 1	Part 2
Policy	0.153*** (0.015)	295.01*** (71.67)	0.207*** (0.019)	303.02** (100.07)	0.158*** (0.016)	-28.67 (43.54)
Post	0.119*** (0.013)	234.43* (119.14)	0.052*** (0.014)	41.33 (204.50)	0.126*** (0.014)	11.04 (66.56)
PolicyXPost	-0.077*** (0.017)	-222.19** (80.21)	-0.044* (0.022)	-339.27** (111.95)	-0.088*** (0.018)	-1.89 (49.41)
PolicyXPost XPhi	0.008 (0.016)	117.44 (89.76)	-0.015 (0.023)	169.76 (119.11)	0.022 (0.017)	40.69 (46.00)
Age	0.007 (0.004)	-36.15 (36.48)	0.011* (0.005)	34.29 (63.44)	0.004 (0.004)	3.82 (17.57)
High School	-0.002 (0.055)	208.53 (204.25)	0.139 (0.083)	-275.08 (398.81)	-0.018 (0.061)	34.98 (108.19)
College	0.020 (0.072)	-754.94 (435.42)	-0.036 (0.079)	-1074.43 (799.43)	0.022 (0.071)	8.18 (109.66)
Marital Status	-0.015 (0.030)	-160.15 (133.54)	-0.031 (0.029)	-12.54 (147.82)	-0.032 (0.03)	-127.91 (110.48)

Children	0.007<br>(0.010)	-17.62<br>(41.57)	0.006<br>(0.012)	-0.59<br>(68.07)	0.005<br>(0.001)	-26.38<br>(19.71)
Residence	0.010<br>(0.038)	-117.38<br>(387.51)	0.086<br>(0.057)	-481.08<br>(932.16)	0.026<br>(0.043)	-72.27<br>(98.41)
House Income	0.000<br>(0.000)	-0.00<br>(0.00)	-0.000<br>(0.000)	-0.00<br>(0.00)	0.000<br>(0.000)	0.00<br>(0.00)
Employment	0.004<br>(0.011)	-27.17<br>(53.90)	0.003<br>(0.013)	-92.48<br>(84.39)	0.009<br>(0.012)	24.40<br>(27.63)
Chronic Diseases	0.001<br>(0.002)	-8.44<br>(15.07)	-0.005<br>(0.003)	-20.01<br>(26.17)	0.001<br>(0.002)	0.90<br>(7.06)
Perceived Health_Good	-0.020*<br>(0.009)	5.72<br>(32.95)	-0.005<br>(0.009)	23.27<br>(57.94)	-0.022*<br>(0.009)	-7.35<br>(21.52)
Perceived Health_Bad	0.018*<br>(0.008)	49.38<br>(36.95)	0.045***<br>(0.010)	60.27<br>(55.95)	0.016*<br>(0.008)	-6.95<br>(21.83)
BMI	0.004**<br>(0.001)	-2.88<br>(5.21)	-0.004*<br>(0.001)	-4.51<br>(6.60)	0.008***<br>(0.002)	-0.85<br>(2.24)
Disablity	-0.029<br>(0.030)	348.19<br>(209.38)	-0.088*<br>(0.034)	623.88*<br>(290.22)	-0.014<br>(0.033)	68.59<br>(63.31)
Year 2012	0.093***<br>(0.009)	17.79<br>(56.98)	0.016<br>(0.009)	-28.15<br>(95.07)	0.099***<br>(0.009)	20.37<br>(30.28)
Year 2013	0.046***	-125.75	-0.007	-36.07	0.047***	8.75

	(0.008)	(92.50)	(0.009)	(155.95)	(0.008)	(48.08)
Year 2014	0.038***	-108.52	0.004	1.11	0.042***	-38.68
	(0.005)	(57.23)	(0.006)	(92.86)	(0.005)	(30.66)
Year 2015	0.029***	-64.46	0.011	-30.37	0.031***	-30.43
	(0.005)	(58.73)	(0.006)	(93.90)	(0.006)	(29.43)
Year 2016	0.036***	46.93	-0.003	-16.67	0.036***	5.07
	(0.005)	(78.68)	(0.006)	(130.02)	(0.005)	(39.02)
_cons	-0.039***	551.01***	-0.004	691.65***	-0.041***	257.91***
	(0.004)	(52.40)	(0.004)	(84.99)	(0.004)	(26.32)
Total	18908	6208	18908	2855	18908	6277

Note. To examine heterogeneity in the effects of the catastrophic coverage expansion between individuals with and without supplemental PHI, we estimated the triple-differences model which augmented the two-part DID regression model by multiplying the interaction term by supplementary PHI status. The coefficient on the interaction term now captures the change in annual total OOP spending on non-covered services attributable to the catastrophic coverage expansion for individuals with supplemental PHI. Coefficients on the triple interaction terms capture differences in the impact of the catastrophic coverage expansion between individual with and without PHI. All models were fully specified. Standard errors are in parentheses.

* $p < 0.05$, ** $p < 0.01$, *** $p < 0.001$.

4. The scope and content of non-covered services remained after 2013

To clarify the scope and content of healthcare services remained non-covered after 2013, we categorize them into three domains: Procedures/Diagnostics, Therapeutic Materials, and Medications as below (Supplemental Table S4). The non-covered services in the table are representative examples, not the comprehensive list.

- **The Procedures/Diagnostics category** includes high-cost imaging (e.g., MRI, CT), genetic testing, and advanced interventional procedures that are often essential for individualized or precision medicine.
- **The Therapeutic Materials category** consists of special-purpose disposable devices such as antibacterial catheters and vascular occlusion tools that help prevent complications and improve clinical outcomes.
- **The Medications category** features high-cost drugs including targeted anticancer therapies and treatments for rare or life-threatening diseases, which posed significant financial burdens before coverage.

< Supplementary Table S4 > Healthcare services remained non-covered after 2013

Procedures/Diagnostics	Therapeutic Materials	Medications
Breast Reconstruction	Indwelling Catheter	Colon cancer treatment (Erbitux)
Auditory Brainstem Transplantation	Urine Drainage Container	Colon cancer treatment (Avastin)
Ocular CT	Antibacterial Indwelling Catheter	Lung cancer Treatemt (Geotrip tablets)
Navigational Guided Procedure	Device for Temporary Vascular Occlusion	Treatment of febrile neutropenia (Neurapex prefilled syringe)
3D Arrhythmia Treatment	Catheter for Removing a Blood Clot	Treatment of febrile neutropenia (Durastine injection prefilled syringe)
8 Genetic Tests for Personalized Cancer Treatment	Proximal Balloon Catheter	Cancer pain medication (Instanil Nasal Spray)

※ **Resource: Ministry of Health and Welfare** (https://www.mohw.go.kr/sotong)

In line with the government's policy to alleviate patients’ financial burden, especially for those with catastrophic conditions, non-covered services were classified based on their necessity, social demand, and cost-effectiveness. Most essential services were fully included in the benefit package, while services deemed non-essential or not sufficiently cost-effective—such as advanced imaging and high-priced cancer drugs—were partially supported by the NHIS. These were planned to be gradually incorporated into full coverage depending on budget availability.

5. Box-Cox Test and Modified Park Test Results for GLM Model Specification

To estimate the DID model, we employed the generalized linear models (GLMs) to account for the skewed distribution of healthcare expenditures. Model specification was guided by Box–Cox and Modified Park diagnostic tests, which inform the choice of link function and variance structure, respectively.

Box–Cox diagnostics indicated log-type mean functions for all expenditure outcomes, with estimated power parameters close to zero for total ($\delta = 0.07$), inpatient ($\delta = 0.17$), and outpatient ($\delta = 0.09$) out-of-pocket spending. Modified Park test results suggested variance–mean relationships consistent with a Gaussian distribution for total and outpatient spending, and modest overdispersion for inpatient spending. These diagnostic results are summarized as below (Supplemental Table S5).

Although Box–Cox diagnostics favored a log link, log-link GLM specifications exhibited convergence instability in this dataset. We therefore employed power link functions as stable approximations to log-type relationships in the conditional expenditure models (power 0.3 for total, 0.2 for inpatient, and 0.5 for outpatient spending), in combination with a Gaussian family.

< Supplementary Table S5 > Model Diagnostic Test Results

Outcomes	Box-Cox δ	Selected Link	Modified Park Coefficient	Family
Total OOP	0.07	Power (0.3)	0.08	Gaussian
Inpatient OOP	0.17	Power (0.2)	0.55	Gaussian
Outpatient OOP	0.09	Power (0.5)	0.09	Gaussian

Note. Box–Cox power parameters close to zero indicate log-type mean functions, but power links were selected as stable approximations to log links where log-link models did not converge. Modified Park coefficients close to zero indicate a Gaussian variance structure.
